# Supplementary material for: Fruits Granola Consumption May Contribute to a Reduced Risk of Cardiovascular Disease in Patients with Stage G2–4 Chronic Kidney Disease
Source: Foods. 2025 Dec 17;14(24):4346. doi: 10.3390/foods14244346 (PMC12733148; doi:10.3390/foods14244346)
Supplement: Supplementary file 1 [file foods-14-04346-s001.zip › foods-4005787-supplementary.pdf]

Questionnaire on Bowel Movements

〇Please describe your bowel movement frequency per week.

Before starting Fruits granola →

One month after starting Fruits granola →

Two months after starting Fruits granola →

〇If you are currently using any constipation drugs, please list them.

Over-the-counter drugs →

Prescription drugs in hospital→
